# Mapping the pathophysiology of schizophrenia: interactions between multiple cellular pathways

**DOI:** 10.3389/fncel.2013.00238

**Published:** 2013-11-28

**Authors:** Chao Deng, Brian Dean

**Affiliations:** ^1^Antipsychotic Research Laboratory, Illawarra Health and Medical Research InstituteWollongong, NSW, Australia; ^2^Centre for Translational Neuroscience, School of Medicine, University of WollongongWollongong, NSW, Australia; ^3^Schizophrenia Research InstituteSydney, NSW, Australia; ^4^Molecular Psychiatry Laboratory, Florey Institute of Neuroscience and Mental HealthParkville, VIC, Australia; ^5^Department of Psychiatry, The University of MelbourneParkville, VIC, Australia

**Keywords:** schizophrenia, dopamine, GABA, acetylcholine, neurodevelopment, glutamate, neuregulin 1, ErbB4

Schizophrenia is a complex disorder involving dysregulation of multiple pathways in its pathophysiology with strong evidence to support roles for dopaminergic, glutamatergic, GABAergic, and cholinergic neurotransmitter systems and their interactions in the pathophysiology of the disorder (Benes, 2009; Karam et al., 2010; Gibbons et al., 2013). Additionally, evidence from genetic, post-mortem and animal studies over the past decade has identified a number of susceptibility factors for schizophrenia, including neuregulin 1 (Nrg1) and its receptor ErbB4, disrupted-in-schizophrenia-1 (DISK1), catechol-O-methyl transferase (COMT), BDNF, and Akt, along with their related pathways, that interact closely with dopaminergic, glutamatergic, and GABAergic neurotransmitter systems (Karam et al., 2010). Hence a key question is how these neurotransmitter systems and their interactions contribute to the pathophysiology of schizophrenia and whether interactive changes in these pathways occur in early brain development, based on the view of schizophrenia as a developmental disorder?

This Frontier Research Topic has brought together leading experts in the field to address these questions from different angles in nine reviews, one theoretic article and two research articles. The first four articles focus on the roles and interactions of neurotransmitters in the pathophysiology of psychiatric disorders. Snyder and Gao (2013) provide an excellent review of NMDA receptor hypofunction hypothesis, suggesting NMDA receptor hypofunction as a convergence point for progression and symptoms of schizophrenia. They also discuss evidence on altered NMDA receptor subunits in schizophrenia and how these alterations interact with multiple schizophrenia susceptibility genes that lead to NMDA receptor dysfunction during development (Snyder and Gao, 2013). Scarr et al. (2013) present an in depth and very detailed coverage of cholinergic involvement in schizophrenia and how it interacts with other neurotransmitters including glutamate, dopamine, GABA and serotonin, as well as its links with the inflammatory/immune system. The review also provides a frame work for testable hypotheses of the potential outcomes of a dysregulated cholinergic system for research into the pathophysiologies of psychiatric disorders (Scarr et al., 2013). Cognitive deficits are considered core symptoms of schizophrenia, while abnormalities in gamma oscillations have been identified in schizophrenia patients that are associated with deficits in attention, working memory, and other cognitive functions (Uhlhaas and Singer, 2010). Furth et al. (2013) reviewed the central role of dopamine D4 receptor in the generation of gamma frequency synchronization of neural networks and cognitive processes via their influence on parvalbumin-expressing GABAergic interneurons. They also examined their close synergistic relationship with neuregulin/ErbB4 signaling, in particular in the prefrontal cortex and hippocampus two major brain regions implicated in schizophrenia (Furth et al., 2013). Furthermore, there has been increasing evidence linking oxidative stress to the pathophysiology of schizophrenia. In their article, Yao and his team reviewed some of these findings, focusing particularly on their findings on homeostatic imbalance of purine catabolism and its association with monoamine neurotransmitters in first episode antipsychotic-naïve patients with schizophrenia (Yao et al., 2013).

In the following three papers, Chana et al. have provided an excellent review of the current progress in the search for biomarkers for schizophrenia and psychosis, focusing on biomarkers for major neurotransmitter systems in post-mortem brain studies, as well as covering some recent and exciting studies in microRNA dysregulation in both the blood and brain of schizophrenia patients (Chana et al., 2013). It is followed by a very timely review of current knowledge on the features of polysialic acid and their synthesizing enzymes (specially ST8SIA2), their functions in regulations of cell adhesion, ion channels, neurotrophins (such as BDNF) and catecholamine neurotransmitters (particularly dopamine), in light of several recent lines of evidence linking polysialic acid to schizophrenia (Sato and Kitajima, 2013). Iwakura and Nawa provide a very clear overview of the ErbB1-4 dependent EGF/neuregulin signals, their role in regulating the development and function of the central nervous system, and the contribution of deficits in ErbB signaling to schizophrenia and neurological disease (Iwakura and Nawa, 2013).

Four articles discuss the animal model of schizophrenia, two of which address the *Nrg1*-cannabinoid interaction in a hypomorphic *Nrg1* (*Nrg1* HET) mouse model of schizophrenia. In an original study, Spencer et al. investigated *Nrg1*-cannabinoid interaction in the hippocampus using proteomics, in which they identified alterations of some proteins involved in vesicular release of neurotransmitters, serotonergic neurotransmission, and growth factor release in response to *Nrg1* hypomorphism and *Nrg1*-cannabinoid interaction (Spencer et al., 2013). In a short review, Karl and Arnold further discussed the complex neuro-behavioral effects of *Nrg1*-cannabinoid interaction and its clinical implications (Karl and Arnold, 2013). In a BDNF heterozygous mouse model, Manning and van den Buuse investigated the effects of chronic methamphetamine treatment during late adolescence/early adulthood on a behavioral endophenotype related to the positive symptoms of schizophrenia, prepulse inhibition (PPI) of the acoustic startle reflex (Manning and van den Buuse, 2013). In the fourth animal model paper, altered dopamine ontogeny in the developmentally vitamin D deficient rat model and its relevance to schizophrenia were reviewed (Kesby et al., 2013). This review suggests that early alterations in dopamine ontogeny are a core feature in the pathophysiology of schizophrenia representing a critical aspect useful to a model of this disease.

Finally, in the context of schizophrenia as a neurodevelopmental disorder, Catts et al. (2013) discuss elegantly the normal development of the prefrontal cortex on the molecular and cellular levels in line with cognitive development, as well as the timing of cognitive decline in schizophrenia. They proposed to reconsider schizophrenia as an outcome from a failure to reach the final state of cortical maturation resulting in retainment of an immature cortex rather than resulting from an excess of adolescent synaptic pruning (Catts et al., 2013).

In summary, these studies illustrate clearly the interactive nature of specific pathways on different levels of the brain from molecular and cellular pathways, and neural circuits to functional deficits contributing to the pathophysiology of schizophrenia. We believe that this Frontier Research Topic will stimulate the development of future collaborative and interdisciplinary research to reveal the unknown mechanisms underlying the pathophysiology of schizophrenia.
